# Targeting the Lactate Transporter MCT1 in Endothelial Cells Inhibits Lactate-Induced HIF-1 Activation and Tumor Angiogenesis

**DOI:** 10.1371/journal.pone.0033418

**Published:** 2012-03-13

**Authors:** Pierre Sonveaux, Tamara Copetti, Christophe J. De Saedeleer, Frédérique Végran, Julien Verrax, Kelly M. Kennedy, Eui Jung Moon, Suveera Dhup, Pierre Danhier, Françoise Frérart, Bernard Gallez, Anthony Ribeiro, Carine Michiels, Mark W. Dewhirst, Olivier Feron

**Affiliations:** 1 Pole of Pharmacology, Institute of Experimental and Clinical Research (IREC), Université catholique de Louvain (UCL), Brussels, Belgium; 2 Department of Radiation Oncology, Duke University Medical Center, Durham, North Carolina, United States of America; 3 Nuclear Magnetic Resonance Research Group, Louvain Drug Research Institute (LDRI), Université catholique de Louvain (UCL), Brussels, Belgium; 4 Department of Pathology, Duke University Medical Center, Durham, North Carolina, United States of America; 5 Department of Radiology and Biochemistry, Duke University Medical Center, Durham, North Carolina, United States of America; 6 Laboratory of Biochemistry and Cellular Biology, University of Namur (FUNDP), Namur, Belgium; Institut Gustave Roussy, France

## Abstract

Switching to a glycolytic metabolism is a rapid adaptation of tumor cells to hypoxia. Although this metabolic conversion may primarily represent a rescue pathway to meet the bioenergetic and biosynthetic demands of proliferating tumor cells, it also creates a gradient of lactate that mirrors the gradient of oxygen in tumors. More than a metabolic waste, the lactate anion is known to participate to cancer aggressiveness, in part through activation of the hypoxia-inducible factor-1 (HIF-1) pathway in tumor cells. Whether lactate may also directly favor HIF-1 activation in endothelial cells (ECs) thereby offering a new druggable option to block angiogenesis is however an unanswered question. In this study, we therefore focused on the role in ECs of monocarboxylate transporter 1 (MCT1) that we previously identified to be the main facilitator of lactate uptake in cancer cells. We found that blockade of lactate influx into ECs led to inhibition of HIF-1-dependent angiogenesis. Our demonstration is based on the unprecedented characterization of lactate-induced HIF-1 activation in normoxic ECs and the consecutive increase in vascular endothelial growth factor receptor 2 (VEGFR2) and basic fibroblast growth factor (bFGF) expression. Furthermore, using a variety of functional assays including endothelial cell migration and tubulogenesis together with *in vivo* imaging of tumor angiogenesis through intravital microscopy and immunohistochemistry, we documented that MCT1 blockers could act as *bona fide* HIF-1 inhibitors leading to anti-angiogenic effects. Together with the previous demonstration of MCT1 being a key regulator of lactate exchange between tumor cells, the current study identifies MCT1 inhibition as a therapeutic modality combining antimetabolic and anti-angiogenic activities.

## Introduction

Solid tumors most often exhaust their oxygen supply creating tumor hypoxia [1]. Hypoxia initially triggers a key metabolic adaptation, the glycolytic switch, during which glycolysis is uncoupled from the TCA cycle and becomes the primary source of ATP production. This process is extremely fast as it initially proceeds through inhibition of the Pasteur effect, a negative feedback exerted by energy metabolites on the glycolytic flux [2]. Glycolysis is rather inefficient at producing energy, yielding only 2 ATP molecules per molecule of glucose whereas full glucose oxidation provides up to 38 ATP. Further adaptations are thus needed to support cell survival and proliferation under sustained (or repeated episodes of) hypoxia. Two main biological paths have evolved. On the one hand, tumor cells exhibit an accelerated glycolytic flux to meet their high bioenergetic and biosynthetic demands independently of oxygen but at the cost of high glucose consumption and lactate release [3]–[5]. On the other hand, the angiogenic switch, corresponding to the initiation of vascular extension, aims at increasing oxygen and nutrient supply to hypoxic tumor sites [6]. These two adaptations require *de novo* protein synthesis and share the transcription factor HIF-1 as a master regulator.

HIF-1 is an αβ-heterodimer: the HIF-1β subunit is constitutively nuclear whereas HIF-1α is inducible by hypoxia [7]. Regulation of HIF-1α protein level involves its posttranslational hydroxylation at proline residues 402 and 564 (human sequence) by prolylhydroxylases (PHDs), predominantly PHD2 [8]. PHDs are Fe(II)- and 2-oxoglutarate-dependent dioxygenases that have an absolute requirement for molecular oxygen [9]. In oxygenated cells, proline hydroxylations target HIF-1α to the von Hippel-Lindau protein complex for poly-ubiquitylation followed by proteasome-dependent degradation [10]. Under hypoxia, HIF PHDs are inactivated, HIF-1α escapes proteolytic degradation, migrates to the cell nucleus, and binds to HIF-1β and co-factors to initiate the transcription of target genes. These target genes include glucose transporters, most glycolytic enzymes, the lactate transporter monocarboxylate transporter 4 (MCT4) and key pro-angiogenic effectors such as vascular endothelial growth factor (VEGF) [11], [12]. Because HIF-1 promotes both glycolytic energy production and angiogenesis, it is not surprising that increased levels of HIF-1α are associated with poor cancer prognosis [13], [14].

Several pieces of evidence suggest that the glycolytic switch triggers angiogenesis [15]. The most direct coupling is probably exerted by lactate, the end-product of glycolysis. Lactate has been known for many years to promote angiogenesis in wounds. It promotes collagen deposition [16], [17] and enhances VEGF production and activity in fibroblasts [18] and macrophages [19]. The underlying mechanism has been proposed to involve lactate oxidation to pyruvate, together with a decrease in NAD^+^ and a subsequent decrease in protein poly-ADP-ribosylation [17]–[20]. In glioma tumor cells, lactate was further shown to trigger HIF-1 activation in a hypoxia-independent manner through inhibition of HIF-1α proline hydroxylations [21], [22], resulting in increased VEGF production by tumor cells.

A direct impact of lactate on HIF-1α expression in endothelial cells has however not been documented. We recently reported that lactate could stimulate nuclear factor-kappa B (NF-κB) activation and interleukin-8 (IL-8) production in ECs [23], proving that these cells could act as a lactate signaling platform continuously fueled by the elevated lactate concentration (1–40 mM) present in the tumor microenvironment [24]. In this study, we report that lactate stimulates HIF-1 activity in ECs, which directly accounts for angiogenesis independently of any additional pro-angiogenic stimulus. We further identify the lactate transporter monocarboxylate transporter 1 (MCT1) as the main regulator of HIF-1 activation by lactate in ECs, and provide unprecedented evidence that HIF-1 inhibition and the associated blockade of angiogenesis may be obtained through MCT1 inhibition.

## Results

### ECs take up exogenous lactate

**Figure 1:**
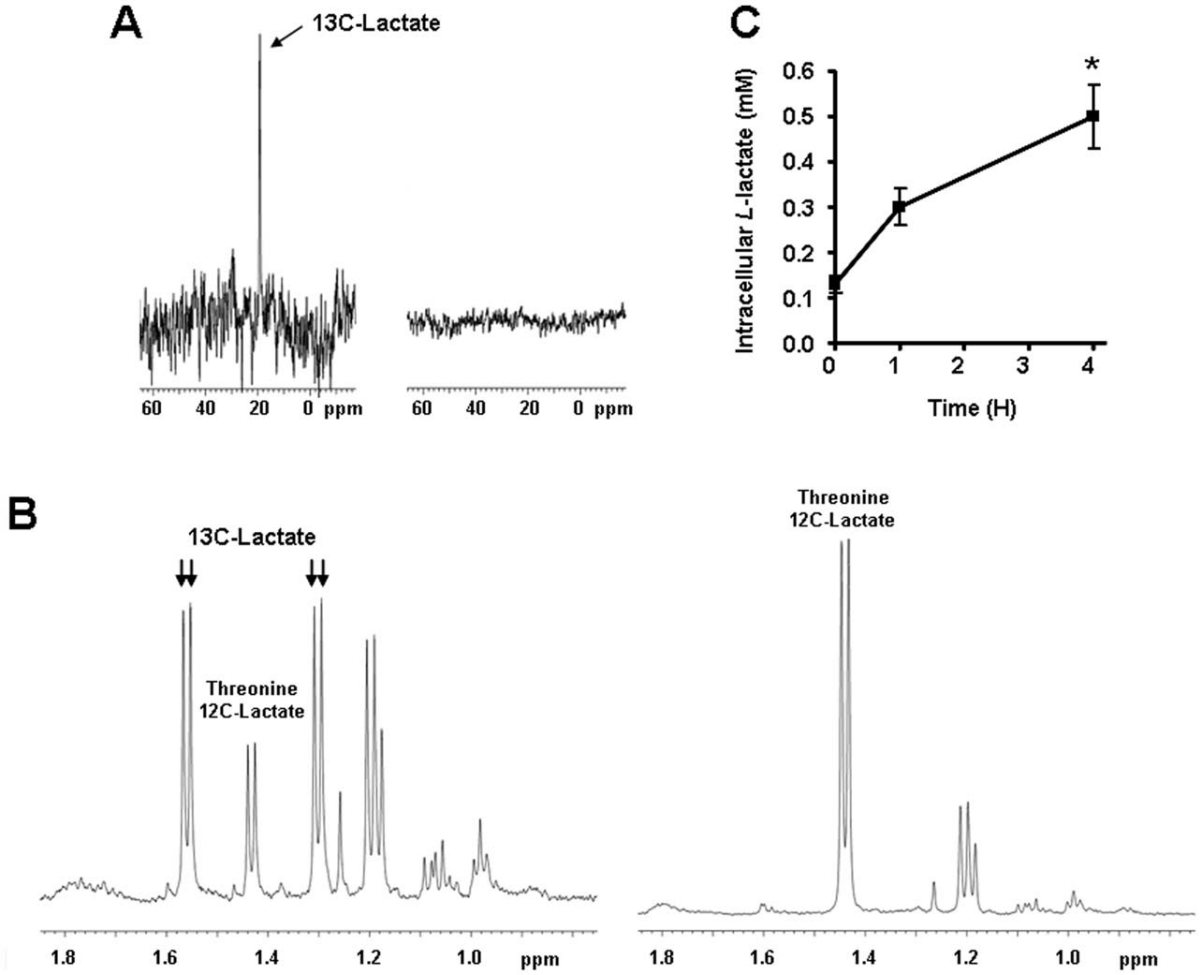
Endothelial cells import exogenous lactate. (A and B) HUVECs were incubated during 6-h with 10 mM of ^13^C-methy-labeled sodium lactate (left panels) or 10 mM of ^12^C sodium lactate (right panels). ^13^C-lactate was detected using nuclear magnetic resonance (NMR) in cell lysates. (A) In ^13^C NMR spectra, a ^13^C-Lactate peak is detected at 19 ppm. (B) In ^1^H NMR spectra, the methyl doublets at 1.56 and 1.30 ppm arise from ^13^C-lactate whereas the doublet at 1.43 ppm arises from a coincidental overlap of endogenous ^12^C-Lactate and threonine. (C) HUVECs were incubated with 10 mM sodium *L*-lactate. *L*-Lactate was detected using an enzymatic assay in the lysate of cells collected at different time points. *p<0.05; *n* = 4. Error bars reflect mean ± SEM.

Confluent ECs were exposed to 10 mM sodium *L*-lactate, a concentration that corresponds to the level of lactate most commonly detected in human tumors [25]. ^13^C-lactate (methyl-labeled) was used in nuclear magnetic resonance (NMR) experiments. ^13^C NMR spectra of cell lysate collected 6-h after treatment showed a peak at 19 ppm corresponding to intracellular ^13^C-lactate (Figure 1A). In the same conditions, ^1^H NMR spectra showed doublets arising from intracellular ^13^C-lactate at 1.56 and 1.30 ppm, thus confirming that ECs import exogenous lactate (Figure 1B). The uptake rate was quantified in an enzymatic assay specific for *L*-Lactate. Kinetic analyses revealed a time-dependent increase in intracellular lactate after exposure to 10 mM extracellular lactate (Figure 1C).

### Exogenous lactate activates HIF-1 in normoxic ECs

**Figure 2:**
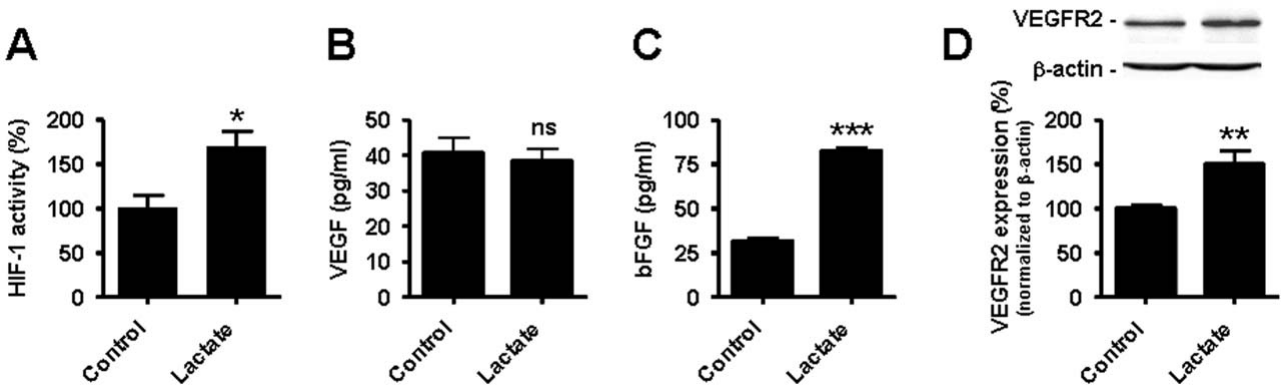
Lactate activates HIF-1 in normoxic endothelial cells. HUVECs were incubated during 24-h with 10 mM lactate or not. (A) The transcriptional activity of HIF-1 was determined using a dual luciferase reporter assay in HUVECs. *p = 0.0207; *n* = 4. (B) VEGF protein level was measured using ELISA in the supernatant of cells. *ns*, non-significant; *n* = 6. (C) bFGF protein level was measured using ELISA in the supernatant of cells. ***p<0.0001; *n* = 5. (D) VEGFR2 protein expression was detected by western blot in cell lysates. **p = 0.0084; *n* = 4–5. (A–D) Error bars reflect mean ± SEM.

Lactate is known to fuel oxidative phosphorylation in tumor cells and favors cell proliferation even in the absence of an exogenous source of serum or growth factors [26]. In similar serum-free conditions, however, lactate cannot rescue glucose-deprived ECs as documented by an accelerated increase in cell death (Figure S1A). In the presence of glucose, it did not modulate the oxygen consumption rate (Figure S1B). In the following experiments, we therefore examined the signaling effects of lactate added to the growth medium of ECs and we focused on HIF-1, based on previous reports in tumor cells [21], [22]. Using a dual luciferase reporter assay in normoxic ECs, we found increased HIF-1 activity after a 24-h treatment with 10 mM lactate (Figure 2A). Consistent with HIF-1 activation, we evidenced the nuclear translocation of HIF-1α (Figure S2). VEGF and basic fibroblast growth factor (bFGF) are major pro-angiogenic agents [27]. VEGF-A is encoded by a HIF-1 target gene [28] while bFGF expression is indirectly induced by HIF-1 [29]. To further investigate HIF-1 activation by lactate, we probed changes in VEGF-A and bFGF release in the supernatant of confluent human umbilical vein endothelial cells (HUVECs) using ELISA. VEGF-A levels were unchanged (Figure 2B) but a 3-fold increase in bFGF secretion was detected after a 24-h incubation with 10 mM lactate (Figure 2C). The same treatment significantly increased the protein expression of VEGF receptor-2 (VEGFR2/flk-1) (Figure 2D), the major transducer of VEGF-mediated angiogenesis in ECs [27]. Increased membrane VEGFR2 expression was confirmed using specific immunostaining in HUVECs (Figure S3A). PCR arrays further revealed that lactate upregulated the transcription of 31 genes in HUVECs by at least ∼2-fold, of which 10 are known to be direct HIF-1 target genes (Table S1).

**Figure 3:**
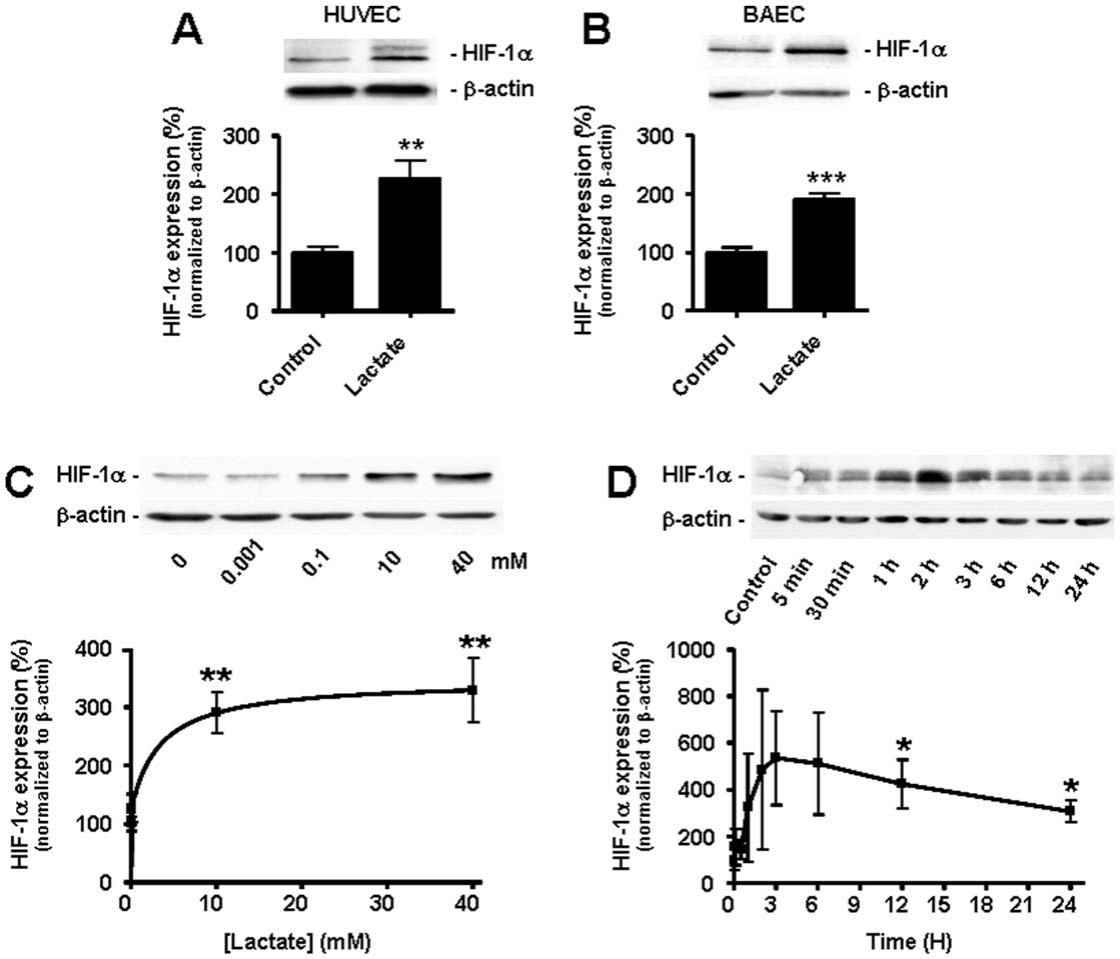
Lactate induces an increase in HIF-1α protein expression in normoxic endothelial cells. HIF-1α protein expression was detected by western blot in normoxic ECs. (A) HUVECs were incubated during 24-h with 10 mM lactate or not. **p = 0.0014; *n* = 8. (B) as in (A) but with BAECs. ***p<0.0001; *n* = 16–17. (C) BAECs were incubated during 24-h with the indicated concentrations of lactate. **p<0.01; *n* = 4. (D) BAECs were incubated during the indicated times with 10 mM of lactate. *p<0.05; *n* = 5–12. (A–D) Error bars reflect mean ± SEM.

HIF-1 activity primarily depends on the stable expression of its α subunit [7]. In normoxic HUVECs, lactate (10 mM) induced a significant increase in HIF-1α protein expression 24-h after treatment (Figure 3A). Similar induction was observed in bovine aortic ECs (BAECs) (Figure 3B). Induction of HIF-1α by lactate was concentration-dependent with a plateau corresponding to a 3-fold increase in HIF-1α protein expression reached after a 24-h exposure to lactate concentrations ≥10 mM (Figure 3C). It was also time-dependent with a 5-fold induction peak at 3-h followed by a slow decrease (Figure 3D).

**Figure 4:**
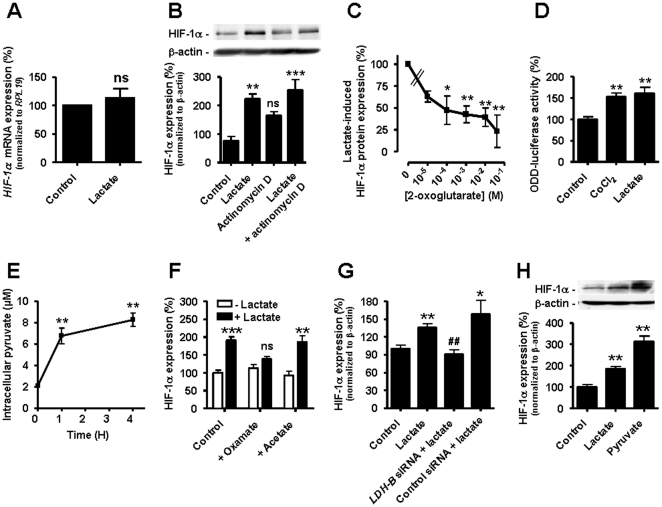
Lactate triggers HIF-1 induction in normoxic endothelial cells. Lactate was used at 10 mM for 24-h. (A) *HIF-1α* mRNA was detected using qRT-PCR in HUVECs incubated with lactate. *ns*, non-significant; *n* = 4. (B and C). HIF-1α protein expression was detected by western blot in BAECs incubated with lactate and/or (B) 5 µg/ml Actinomycin D (**p<0.01, ***p<0.005, *ns*, non-significant compared to control, *n* = 3–4); (C) increasing concentrations of 2-oxoglutarate. *p<0.05, **p<0.01; *n* = 5–6. (D) ODD-driven luciferase activity was assessed in HUVECs incubated with 150 µM CoCl_2_ or lactate. **p<0.01; *n* = 5. (E) Intracellular pyruvate was detected using an enzymatic assay in HUVECs incubated with lactate. **p<0.01; *n* = 4. (F–H) HIF-1α protein expression was detected by western blot. (F) BAECs were incubated with lactate, oxamate (10 mM), and/or acetate (10 mM). **p<0.01, ***p<0.005, *ns*, non-significant compared to control cells without lactate; *n* = 4–16. (G) HUVECs transfected with the indicated siRNA were incubated with lactate. *p = 0.0170, **p = 0.0019 compared to control; ^##^ *p* = 0.0056 compared to lactate alone; *n* = 3–9. (H) BAECs were incubated with pyruvate (10 mM), lactate, or not. **p<0.01 compared to control; *n* = 3–19. (A–H) Error bars reflect mean ± SEM.

The canonical regulation of HIF-1 activity involves the posttranslational hydroxylation of HIF-1α on 2 proline residues, a reaction catalyzed by HIF PHDs that promotes HIF-1α degradation [8]. Our observations that lactate did not significantly alter HIF-1α transcription (Figure 4A) and still induced HIF-1α protein expression in the presence of the transcription inhibitor Actinomycin D (Figure 4B) prompted us to check whether lactate could interfere with the PHD reaction. Considering that PHD activity requires 2-oxoglutarate as a substrate and may therefore be influenced by other carboxylates [21]–[23], we designed competition experiments between lactate and 2-oxoglutarate in normoxic ECs. Addition of 2-oxoglutarate to ECs concentration-dependently reduced the abundance of HIF-1α in ECs exposed to lactate (Figure 4C; a representative blot is shown in Figure S4A). We also used an ODD-Luc construct to detect PHD activity [30] and showed that 10 mM lactate triggered ODD-driven luciferase activity as efficiently as 150 µM CoCl_2_ (Figure 4D).

We next aimed to determine whether lactate or pyruvate (resulting from intracellular lactate oxidation) exerted these effects on PHD activity. Pyruvate cannot be detected using NMR. Therefore, we developed an enzymatic assay to show that exposure of ECs to exogenous lactate led to a time-dependent increase in intracellular pyruvate (Figure 4E). Lactate oxidation to pyruvate is preferentially catalyzed by the lactate dehydrogenase-B (LDH-B) gene product. We found that the LDH competitor oxamate [31] actually blocked lactate-induced HIF-1α protein expression in normoxic ECs, a property which was not shared by the structurally-related monocarboxylate acetate (Figure 4F, a representative blot is shown in Figure S4B). Oxamate inhibits both LDH-A and LDH-B. To confirm the involvement of LDH-B, *LDH-B* was silenced with a specific siRNA (see Figure S5A). This also prevented lactate-induced HIF-1α expression (Figure 4G and Figure S4C). Finally, we also provided evidence that pyruvate was a more potent inducer of HIF-1α than lactate in normoxic ECs (Figure 4H).

### Targeting MCT1 blocks lactate-induced HIF-1 activation in normoxic ECs

**Figure 5:**
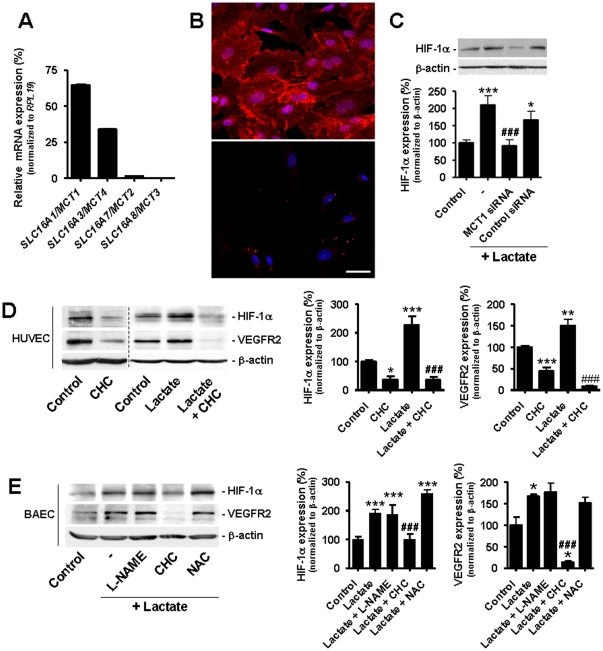
Targeting MCT1 inhibits lactate-induced HIF-1 activation in normoxic endothelial cells. (A) Relative mRNA expression was determined in confluent HUVECs using quantitative RT-PCR. *n* = 3. Error bars reflect mean ± SEM. (B) Immunostaining showing the membrane expression of MCT1 in HUVECs (upper panel) and negative control (lower panel, omission of primary antibody). Bar = 40 µm. (C) HUVECs transfected with the indicated siRNA were incubated during 24-h with 10 mM lactate or not. HIF-1α protein expression was detected by western blot. *p<0.05, ***p<0.005 compared to control; ^###^ *p*<0.005 compared lactate alone; *n* = 6–14. (D and E) HIF-1α and VEGFR2 protein expression was detected by western blot. (D) HUVECs were incubated during 24-h with 10 mM lactate and/or 5 mM α-cyano-4-hydroxycinnamate (CHC). *p<0.05, **p<0.01, ***p<0.005 compared to control; ^###^ *p*<0.005 compared to lactate alone; *n* = 3–12. (E) BAECs were incubated during 24-h with 10 mM lactate, 5 mM *N* ^ω^-nitro-*L*-arginine methyl ester (L-NAME), 5 mM α-cyano-4-hydroxycinnamate (CHC), and/or 5 mM *N*-acetyl-*L*-cysteine (NAC). *p<0.01, ***p<0.005 compared to control; ^###^ *p*<0.005 compared to lactate alone; *n* = 3–14. (C–E) Error bars reflect mean ± SEM.

The lactate anion can shuttle between extracellular and intracellular compartments through monocarboxylate transporters (MCTs) among which MCT subtypes 1–4 function as passive lactate-proton symporters [32], [33]. Using quantitative PCR to compare mRNA levels of these 4 transporters, we identified *MCT1* as the main transcript in HUVECs (Figure 5A). The corresponding protein was localized at the plasma membrane of the cells, as shown by immunocytochemistry in Figure 5B and in Figure S3B where MCT1, its anchor protein basigin/CD147 and the endothelial marker CD31 colocalized. Because MCT1 mediates the uptake of lactate in several cell types [26], [32], [33], we checked whether MCT1 was involved in lactate signaling in ECs. Silencing MCT1 with a specific siRNA (see Figure S5B for target extinction) prevented HIF-1α protein stabilization in HUVECs incubated with lactate (10 mM) for 24 h (Figure 5C). Consistent with the involvement of MCT1, α-cyano-4-hydroxycinnamate (CHC), a reversible inhibitor that has a 10-fold selectivity for MCT1 compared to other MCTs [34], prevented lactate-induced HIF-1α protein expression not only in HUVECs (Figure 5D) but also in BAECs (Figure 5E) without showing intrinsic EC toxicity (Figure S6). These results were verified using a series of known MCT1 inhibitors that all successfully blocked lactate-induced HIF-1α protein stabilization in BAECs (Figure S7). The functional consequence of MCT1 inhibition was addressed by measuring changes in VEGFR2 expression. CHC blocked lactate-induced upregulation of VEGFR2 protein expression in HUVECs (Figure 5D) and in BAECs (Figure 5E). It also reduced basal HIF-1α and VEGFR2 protein expressions in HUVECs (Figure 5D). We ruled out a major contribution of two known HIF-1 activators, nitric oxide (NO) and reactive oxygen species (ROS) [30], in our experimental conditions. Lactate-driven HIF-1α and VEGFR2 protein expressions were indeed resistant to the NO synthase inhibitor *N* ^ω^-nitro-*L*-arginine methyl ester (L-NAME) and to the general antioxidant *N*-acetyl-*L*-cysteine (NAC) (Figure 5E).

### Targeting MCT1 inhibits lactate-induced angiogenesis in tumors

**Figure 6:**
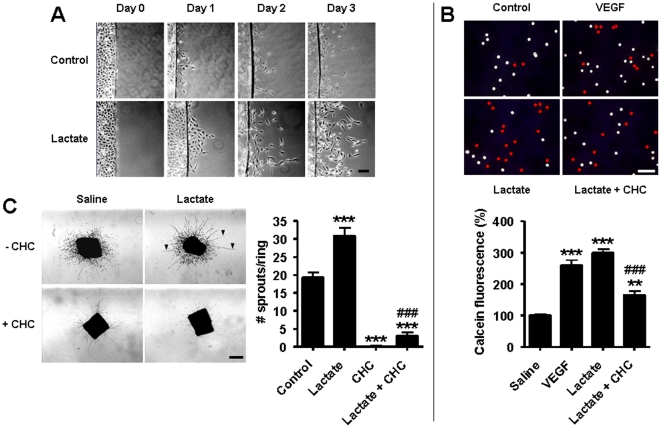
Targeting MCT1 inhibits lactate-induced endothelial cell migration and vascular sprouting. (A) Confluent BAECs on coverslips were placed into cell-free dishes in the presence of 10 mM lactate or not. Pictures show cell migration over time. Bar = 50 µm. (B) Quantification of EC migration was performed in a transwell assay in which calcein-labeled BAECs migrated during 24-h through a Matrigel-coated membrane towards serum-free medium containing 10 ng/ml VEGF, 10 mM lactate, or lactate and 5 mM CHC. Pictures show the membranes where pores occupied by endothelial cells (detected using a caveolin-1 antibody) are labeled with a red bullet. Bar = 50 µm. The graph shows calcein fluorescence in the bottom well. **p<0.01, ***p<0.005 compared to control; ^###^ *p*<0.005 compared to lactate; *n* = 3. (C) Consecutive rings of a mouse aorta were cultured during 10 days in collagen gels containing 10 mM lactate and/or 2 mM CHC. Pictures show endothelial sproutings (arrowhead point at typical linear structures) and fibroblast seeding (scattered cells). Bar = 500 µm. The graph shows the number of sprouts per ring. ***p<0.005 compared to control; ^###^ *p*<0.005 compared to lactate; *n* = 6–11. (B and C) Error bars reflect mean ± SEM.

We next investigated whether lactate-induced HIF-1 activation conferred an angiogenic phenotype to ECs. We first observed that lactate increased EC motility, as illustrated by the increased capacity of confluent ECs to colonize a cell-free area in the presence of lactate compared to vehicle (Figure 6A). Lactate-induced migration was quantified in transwell experiments in which ECs in the upper chamber had to invade a layer of Matrigel matrix to reach the bottom well containing lactate. In this assay, 10 mM lactate promoted EC migration as efficiently as 10 ng/ml VEGF (Figure 6B). EC migration was partly inhibited by CHC, indicating that lactate uptake is as an integral part of the migratory phenotype. The involvement of MCT1 in lactate-induced endothelial invasion was further tested *ex vivo*. A 3D-culture of aortic rings in collagen gels was used to evaluate the outgrowth of linear endothelial structures from the preexisting vessel [35]. Both basal and lactate-induced endothelial responses were completely blocked by CHC (Figure 6C). The inhibition was sustained but reversible, as sprouting eventually resumed 14 days after the CHC treatment.

**Figure 7:**
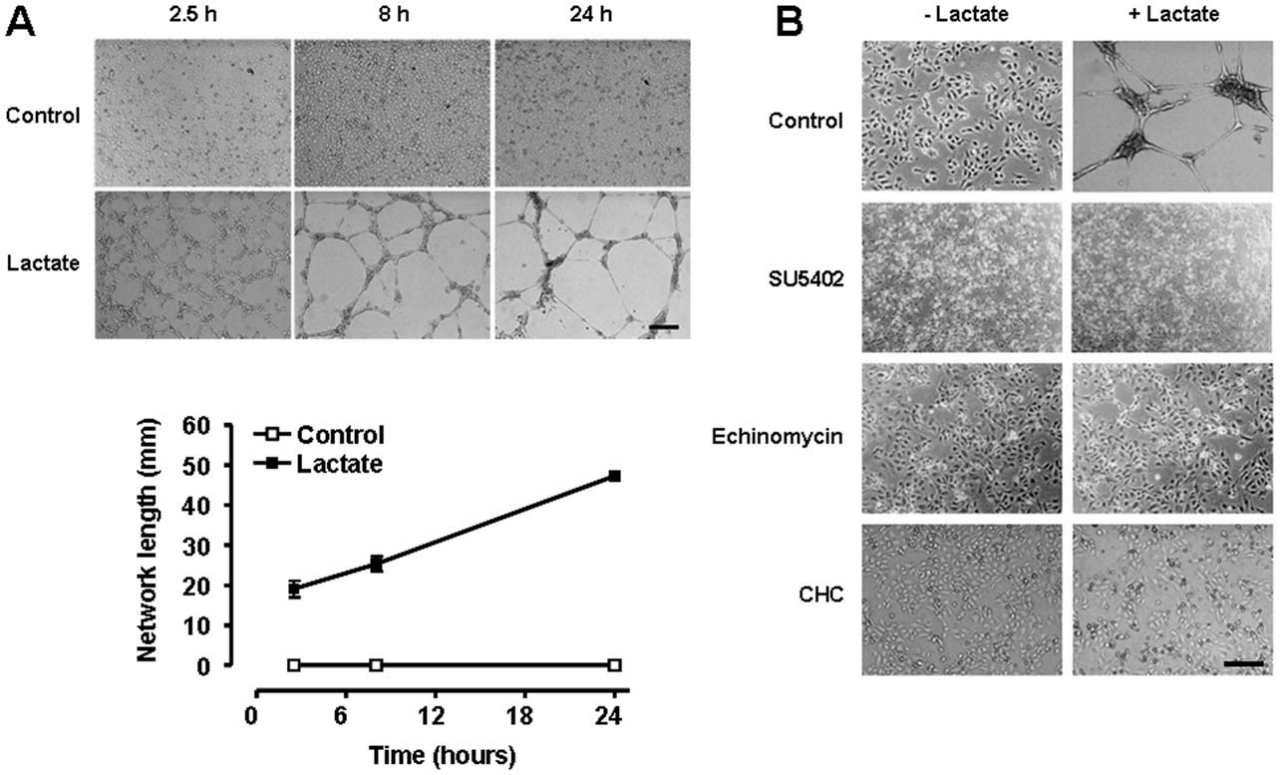
MCT1 inhibition blocks lactate-induced endothelial tube formation. HUVECs were seeded on growth factor-reduced Matrigel containing 10 mM lactate or not and monitored for their ability to generate vascular tubes. (A) Pictures of the upper panel show endothelial network formation over time (bar = 500 µm), which was quantified in the lower graph. *n* = 4. Error bars reflect mean ± SEM and are sometimes smaller than symbols. (B) The tube formation assay was performed in the presence of 10 µM SU5402 (targeting VEGF and bFGF receptors), 10 nM echinomycin (targeting the transcriptional activity of HIF-1), or 2 mM of CHC (targeting MCT1). Pictures were captured after 8-h of culture in the Matrigel matrix. Bar = 200 µm.

The angiogenic conversion of ECs was also assayed on Matrigel [36]. In this assay, lactate alone promoted sustained endothelial tube formation (Figure 7A), a response that was completely inhibited using SU5402 (targeting VEGF and bFGF receptors), echinomycin (an inhibitor of the transcriptional activity of HIF-1), and the MCT1 inhibitor CHC (Figure 7B). Using undiluted Matrigel, we demonstrated that MCT1 silencing with a specific shRNA not only inhibited lactate-induced tubulogenesis but also basal tubulogenesis (Figure S8), suggesting autocrine lactate signaling in cultured ECs.

**Figure 8:**
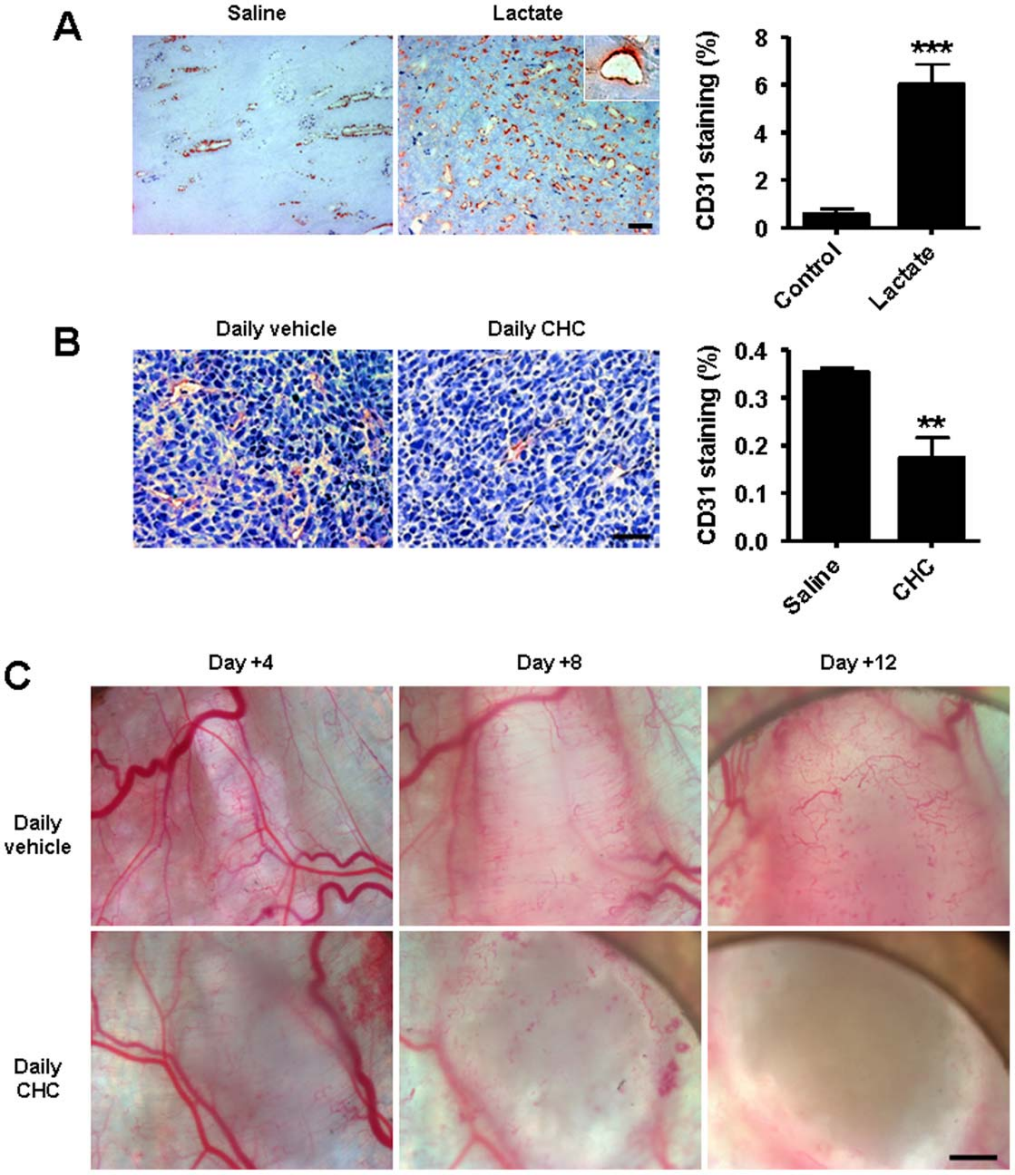
MCT1 inhibition blocks tumor angiogenesis. (A) Two Matrigel plugs were implanted subcutaneously in the same mouse, one containing 10 mM lactate (left flank) and the other one an equal volume of saline (right flank). Plugs were microdissected 7 days after implantation. Pictures show CD31 immunostainings (bar = 100 µm; inset: a vascular structure carrying red blood cells), which were quantified in the graph. ***p = 0.0007; *n* = 4. Error bars reflect mean ± SEM. (B and C) On Day 0, mice were randomly assigned to treatment groups consisting of daily i.p. injections of CHC (1 mmol/Kg) or vehicle. (B) Mice carrying LLc tumors (4±1 mm) were treated. Pictures show CD31 immunostainings of size-matched tumors (bar = 20 µm), which were quantified in the graph. **p = 0.0051; *n* = 4. Error bars reflect mean ± SEM. (C) Intravital microscopy pictures were captured over time in treated mice carrying tumors generated with MCT1-negative TLT tumor cells. Bar = 1 mm.

We performed *in vivo* experiments to validate lactate as a pro-angiogenic agent and MCT1 as a new anti-angiogenic target. First, Matrigel plugs were implanted subcutaneously in each flank of mice, the left plug containing 10 mM lactate and the right plug an equal volume of saline. Seven days later, CD31 immunolabeling of collected plugs revealed a ∼10-fold increase in the endothelial colonization of lactate-containing plugs compared to controls (Figure 8A). Most vascular structures adopted a circular shape and were perfused (Figure 8A, inset), thus confirming lactate as a pro-angiogenic agent. In a second set of experiments, we tested the anti-angiogenic activity of CHC in tumors. Comparison of size-matched mouse Lewis Lung carcinoma (LLc) tumors showed that chronic MCT1 inhibition reduced the endothelial density at vascular hotspots by ∼2-fold compared to vehicle treatment (Figure 8B). In this model, CHC may interfere with the metabolic activity of MCT1-expressing tumor cells [26]. To better discriminate between direct and indirect anti-angiogenic effects, we repeated the experiment using MCT1-negative hepatocarcinoma (TLT) cells that were previously shown to be insensitive to the metabolic regulation by CHC [26]. Intravital microscopy revealed that chronic MCT1 inhibition completely prevented tumor angiogenesis in this model (Figure 8C). While new vascular structures continuously developed during the growth of saline-treated tumors, the angiogenic phenotype was lost in response to CHC administration (see pictures at day +12). Hemorrhagic foci were already observed after 8-days of treatment.

## Discussion

The two main findings of our study are (i) that lactate stimulates HIF-1 activity directly in normoxic ECs resulting in the increased expression of relevant pro-angiogenic targets including the prototypical VEGF receptor VEGFR2, and (ii) that the anti-angiogenic effects of MCT1 inhibition include inhibition of HIF-1 activation in ECs. This activity was initially identified *in vitro* using two different EC lines in which MCT1 was targeted pharmacologically or using RNA interference. It was confirmed *in vivo* by using the experimental MCT1 inhibitor CHC in two different tumor types.

**Figure 9:**
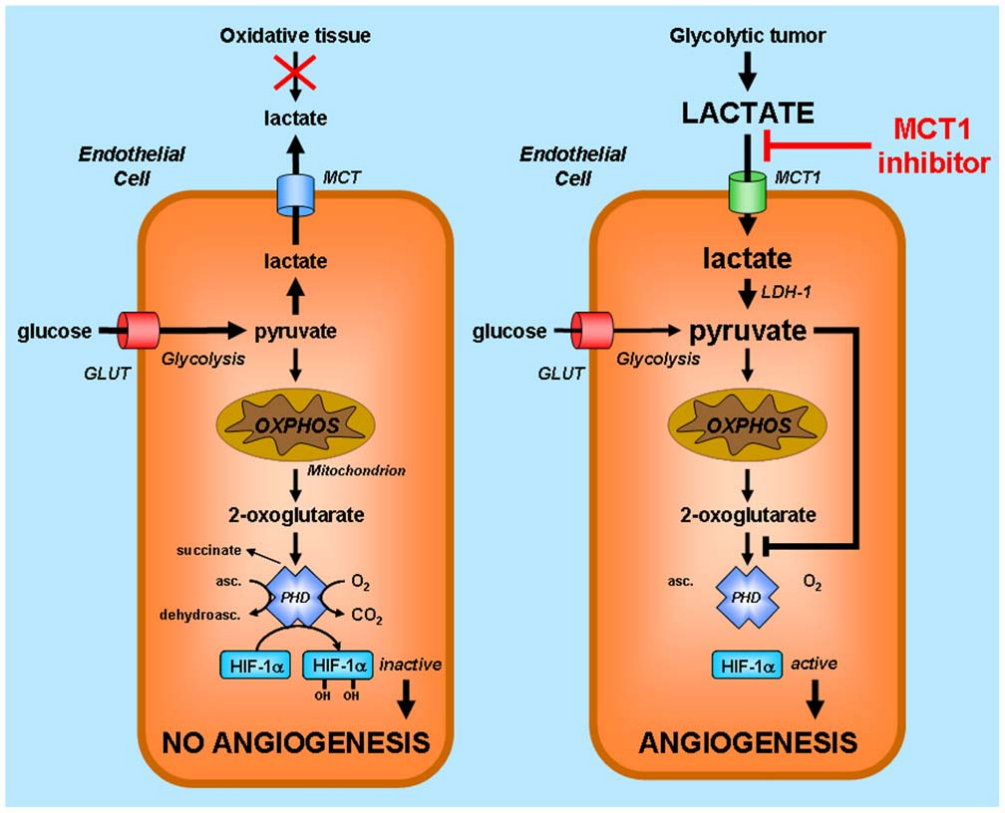
Model illustrating the anti-angiogenic activity of MCT1 inhibitors targeting lactate-induced HIF-1 activation in endothelial cells. HIF-1 activity primarily depends on the stability of HIF-1α. When HIF prolylhydroxylase (PHD) is active as it is the case for normoxic endothelial cells (ECs) in oxidative tissues (*left*), HIF-1α is hydroxylated and addressed to the proteasome for degradation. PHD requires 2-oxoglutarate as a substrate and is competitively inhibited by pyruvate. Normoxic ECs in glycolytic tissues such as tumors (*right*) take up exogenous lactate originating from distant hypoxic cells, a process under the control of monocarboxylate transporter 1 (MCT1). ECs do not primarily use lactate as a fuel for oxidative phosphorylation (OXPHOS). Lactate oxidation by LDH-B rather increases the pool of intracellular pyruvate, now available to compete with 2-oxoglutarate from PHD, resulting in HIF-1α protein stabilization, HIF-1 activation, and angiogenesis. Because MCT1 is the main transporter for lactate uptake by ECs, targeting MCT1 inhibits lactate-induced angiogenesis in tumors. Abbreviations: asc., ascorbate; dehydroasc., dehydroascorbate; GLUT, glucose transporter.

Our demonstration is based on the unprecedented identification in ECs of a lactate-signaling pathway leading to HIF-1 activation under normoxia. This pathway involves lactate uptake, the conversion of lactate to pyruvate by LDH-B, a competition between pyruvate and 2-oxoglutarate supporting a pyruvate-mediated inhibition of PHD2, and, consequently, HIF-1 activation independently of hypoxia (Figure 9). Lactate did not increase the oxygen consumption rate of ECs (see Figure S1B), ruling out a contribution of metabolic hypoxia to HIF-1 activation. A similar effect of monocarboxylates including pyruvate and lactate has been previously described in malignant cells by Lu et al. [21], [22]. Final effectors are however different. While lactate was shown to promote VEGF production by tumor cells [22], we show here that in ECs, it upregulates VEGFR2 expression without modulating VEGF protein secretion despite increased *VEGF-A* gene transcription. Because VEGFR2 is the main transducer of the pro-angiogenic effects of VEGF [27], one can logically propose that, in complex tumor tissues, lactate exquisitely stimulates VEGF signaling by acting on both the growth factor and its cognate receptor. This paradigm is further supported by the influence of lactate on stromal cells. Lactate indeed promotes VEGF production by fibroblasts and macrophages [18], [19], and enhances VEGF activity through inhibition of ADP-ribosylation in several cell types [18]–[20]. Of note, the existence of a HIF-1α-driven, VEGF-mediated autocrine loop in ECs could further reinforce the anti-angiogenic effects of MCT1 inhibition in ECs [37], [38]. Finally, it should be emphasized that although the lactate induction of the VEGF/VEGFR2 signaling pathway is independent of hypoxia in the target cells, lactate may arise from anaerobic glycolysis [24] as well as aerobic glycolysis (the Warburg effect) [39], or through the malate shuttle from the oxidative metabolism of glucose and glutamine [40], [41]. Lactate flux rate is likely to be different between “target” cells signaling through lactate *vs* cells consuming or producing lactate. For instance, while lactate-fueled respiration of tumor cells requires high-rate lactate uptake and high MCT1 expression [26], lower MCT1 expression in ECs could efficiently support the low-rate lactate uptake accounting for lactate signaling. Different levels of MCT1 expression could explain why Koukourakis et al. [42] failed to detect MCT1 in the vascular structures of colorectal adenocarcinomas using immunohistochemistry.

Although we focused on the paradigm that HIF-1α-driven VEGFR2 receptor expression is a target of MCT1 inhibition to account for the observed anti-angiogenic effects, inhibition of other pro-angiogenic HIF-1α targets is very likely to support the functional impact of blocking lactate transport in ECs. For instance, we found that bFGF release was increased in response to lactate (Figure 2C) and thus that in the absence of increased VEGF, the inhibition of lactate-induced tubulogenesis on Matrigel with SU4502, a mixed inhibitor of VEGFR2 and fibroblast growth factor receptor 1 (FGFR1) (Figure 7B), strongly suggests that bFGF participates to the pro-angiogenic effects of lactate. Of interest is also the ability of bFGF to stimulate the activity of aminophospholipid translocase in ECs [43], an enzyme required for the maintenance of lipid asymmetry of the membrane, proper membrane anchorage of proteins, endocytosis and membrane recycling [44], [45]. In erythrocytes, GLUT-dependent arabinose transport and MCT-dependent lactate transport were shown to be inhibited by cleavage of either phosphatidylcholine or sphingomyelin [46], stressing out that membrane fluidity and asymmetry are important for the activity of these transporters. Whether lactate-induced bFGF production facilitates the membrane translocation of transporters (such as MCT1) and receptors (such as bFGFR and VEGFR2) involved in the angiogenic phenotype of ECs warrants further investigation.

More generally, previous work indicates that targeting MCT1 offers the possibility to simultaneously inhibit several major pro-angiogenic pathways with a single therapeutic molecule. First, MCT1 inhibition may interfere with the viability of angiogenic-factor-producing-cells including cancer cells, macrophages and fibroblasts; all these cells express MCT1 [26], [42]. Note that the impact may be indirect since MCT1 inhibition in oxidative tumor cells may favor the death of hypoxic glycolytic cells [26], and alteration in the fibroblast capacity to take up lactate and export pyruvate (both through MCT1) in tumors may reduce tumor cell proliferation [42]. Second, as documented in a previous study, MCT1 inhibition can block lactate-induced NF-κB activation in ECs, with a subsequent downregulation of autocrine IL-8 signaling and IL-8-mediated angiogenesis [23]. Interestingly, this latter effect was also in part dependent on a direct competition between 2-oxoglutarate and lactate for PHD2. Given this mode of action, other targets may be anticipated. For example, 2-oxoglutarate is a necessary cofactor for factor inhibiting HIF-1 (FIH-1), a 2-oxoglutarate-dependent dioxygenase that inactivates HIF-1 through HIF-1α asparagine hydroxylation [47]. Because PHDs and FIH-1 are simultaneously inhibited by compounds such as dimethyloxalylglycine that displace 2-oxoglutarate from the active enzyme sites [48], our data suggest that lactate-dependent dioxygenase inhibition may extend to FIH-1. Both PHD2 and FIH-1 also control HIF-2 activity through regulating HIF-2α hydroxylations [49], suggesting that the activity of lactate could further include HIF-2 activation in ECs. HIF-1 and HIF-2 control both different and overlapping sets of genes modulating angiogenesis and vascular stability and maturation, respectively [50]–[53]. Whether the detection of mature blood vessels in lactate-containing Matrigel plugs (Figure 8A) and the apparent antivascular effects of MCT1 inhibition *in vivo* (Figure 8C) are related to changes in endothelial HIF-2 activity remains to be determined. The anti-angiogenic effects of MCT1 inhibitors that we document here offers an additional rationale for the antitumor effects of MCT1 inhibitors that we identified previously in several types of tumors and attributed to metabolic effects [26]. In a model of colon cancer xenograft involving the co-injection of HUVECs, a downregulation of MCT1 expression in ECs was indeed documented to significantly retard tumor growth [23].

In conclusion, we have shown that lactate activates HIF-1 in normoxic ECs and consecutively promotes the expression of bFGF and VEGFR2, the latter reinforcing the stimulation of the VEGF signaling pathway promoted by the lactate-induced VEGF expression occurring in tumor and stromal cells. Importantly, we also report that in ECs, MCT1 inhibition can thus drive direct anti-angiogenic effects through a reduction in HIF-1 activity, which, together with the antimetabolic effects of MCT1 inhibition on tumor cells, considerably enlarges the therapeutic potential of this class of drugs.

## Materials and Methods

### Cells and chemicals

BAECs (Clonetics) were routinely cultured in EBM-EGM (Cambrex), HUVECs (Clonetics) in EBM2-MV (Cambrex), and LLc (LGC Standards, [54]) and TLT (originally obtained at from HS Taper, University of Louvain [55]) tumor cells in DMEM containing 4500 mg/liter glucose (Invitrogen) with 10% FBS at 5% CO_2_ and 21% O_2_, 37°C. Glucose-deprived DMEM was from Krackeler Scientific. Media were supplemented with 1% penicillin-streptomycin. All experiments were performed in pyruvate-free media buffered at pH 7.3 (3.7 g/liter NaHCO_3_). To minimize fluctuations due to unequal growth rates, all assays were performed on confluent cells. Hypoxia (1% O_2_) was achieved as previously described [56]. Cell death was quantified using the NucleoCounter device from ChemoMetec. Unless stated otherwise, all chemicals including CHC were from Sigma. Carboxylates were used as sodium salts.

### Determination of intracellular *L*-lactate and pyruvate

For NMR experiments, HUVECs in EBM2 media were incubated during 6-h to 10 mM ^13^C-methyl-labeled lactate (Isotec, Sigma), washed twice with DPBS, lysed and collected in 0.9 M perchloric acid (1∶10 in diH_2_O). ^12^C-(unlabeled)-lactate was used as control. Lysates were spun at 4°C for 10 min at 12,000 rpm. ^13^C and ^1^H NMR spectra acquisitions were performed on supernatants using a 500-MHz Varian Inova spectrometer equipped with a 5-mm tunable broadband probe. ^13^C-NMR spectra were acquired at 125.7 MHz with 45° flip angle, 0.8-second interpulse delay, a 1.334 s acquisition time, and at a temperature of 25°C. Control runs of known concentrations of lactate were used to assess peak shape and ppm position. VNMRJ software by Varian (Cary, NC) was used to acquire and analyze spectra. All samples were tuned and shimmed before data acquisition. For enzymatic measurements, confluent HUVECs were incubated with 10 mM *L*-lactate, washed twice with PBS containing 10 mM *D*-Lactate (to saturate MCTs), and lysed in 200 µl ice-cold RIPA Buffer (50 mM Tris HCL pH 7.4, 150 mM NaCl, 1% Triton-x 100, 0.05% sodium deoxycolate, 0.1% SDS,1 mM EDTA, 1 mM Na_3_VO_4_ and protease inhibitors). Samples were filtered through centrifugation columns with a 10 KDa cutoff. *L*-Lactate and pyruvate concentrations were quantified in clear cell lysates using specific enzymatic assays on an ISCUS*^flex^* analyzer (CMA Microdialysis AB).

### Oximetry

Determination of cell oxygen consumption rates was carried out using electron paramagnetic resonance (EPR) in DMEM containing 4500 mg/liter glucose, dextran 10% [57]. Briefly, 2×10^7^ viable cells/ml were sealed in glass capillary tubes in the presence of 0.08 mM of the O_2_ sensor 4-oxo-2,2,6,6-tetramethylpiperidine-d-15N-1-oxyl (CDN isotopes) and with 10 mM lactate where indicated. Cells were maintained at 37°C during recording on a Bruker EMX EPR spectrometer operating at 9 GHz.

### Dual luciferase reporter assays

Dual luciferase reporter assays were performed with the dual luciferase kit (DLR) from Promega using pGL3-(PGK-HRE6)-TK-Luc as reporter of HIF-1 activity [10] and pODD-Luc as reporter of PHD activity [30]. Cells were transfected using electroporation (Amaxa Nucleofector, Lonza).

### Western blotting, immunohistochemistry and antibodies

We used previously disclosed protocols for immunocytochemistry and immunohistochemistry [58] except that HUVECs were permeabilized with 0.05% saponin for MCT1 detection. Western blotting (WB) was done as previously shown [59] with the slight modification that proteins were not heat-denaturated for HIF-1α protein detection. This allowed the detection, in whole cell lysates, of basal HIF-1α that would otherwise not be detected [60]. For Figure S2, nuclear extraction was performed according to manufacturer's recommendations using the nuclear extraction kit from Active Motif. Primary antibodies were: a mouse monoclonal against HIF-1α (BD), a rabbit polyclonal raised against an intracellular epitope of MCT1 (AB3538P, Chemicon), a rabbit polyclonal against caveolin-1 (BD), a rabbit polyclonal against laminin A/C (Santa Cruz), a goat polyclonal raised against an epitope comprising an an extracellular domain of VEGFR2 (R&D) for immunohistochemistry, a mouse monoclonal against VEGFR2 (Santa Cruz) for WB, a mouse monoclonal against CD147 (BD), a mouse monoclonal against β-actin (Sigma), a mouse monoclonal against CD31 (Dako) for Figure S3, and a rat monoclonal against CD31 (BD) for Figure 8. For immunohistochemistry, areas of positive staining were quantified using the Framework for Image Dataset Analysis (FRIDA) software developed by Johns Hopkins University.

### ELISA

VEGF-A and bFGF protein levels were measured in the supernatant of confluent HUVECs using the Human VEGF Quantikine ELISA Kit (R&D) and the Human bFGF Quantikine ELISA kit (R&D) according to manufacturer's instructions.

### PCR and RNA interference

The effects of a 24-h exposure of HUVECs to lactate and saline were compared using the Human Angiogenesis RT2 Profiler PCR Array from SABiosciences, according to manufacturer's instructions. In each condition, total mRNA extracted from 3 independent dishes was pooled. To silence *MCT1* and *LDH-B* mRNAs, HUVECs were transfected with specific siRNAs as reported previously [61]. siRNAs (Qiagen) targeted the following sequences: hMCT1 AAGAGGCUGACUUUUCCAAAU, hLDHB AAGAUUGUAGUGGUAACUGCA. Allstar siRNA (Qiagen) was used as negative control. MCT1 shRNA was purchased from Open Biosystems (clone TRCN0000038340); scrambled control shRNA was Addgene plasmid 1864. The following primers were used for SYBR green quantitative PCR [61]: hMCT1 sense, 5′-GTGGCTCAGCTCCGTATTGT-3′, antisense, 5′-GAGCCGACCTAAAAGTGGTG-3′; hMCT2 sense, 5′-CAACACCATTCCAAG-ACAGC-3′, antisense 5′-TGGCTGTTATGTACGCAGGA-3′; hMCT3 sense, 5′- GGATGTGTT-GAAGAACTATGAGATC-3′, antisense 5′-CCGGGTTCCTCTGCAACA-3′; hMCT4 sense, 5′-CAGTTCGAGGTGCTCATGG-3′, antisense, 5′- ATGTAGACGTGGGTCGCAT-3′. Housekeeping human ribosomal protein L19 (hRPL19) primers were sense, 5′-CAAGCGGATTCTCATGGAACA-3′, antisense, 5′-TGGTCAGCCAGGAGCTTCTT-3′.

### Endothelial cell migration assays

To image EC migration, we used an assay analogous to a scratch injury test [62]. BAECs were cultured to confluence on gelatin-coated circular coverslips, which were then transferred to adhere onto 100 mm gelatin-coated dishes containing EBM-EGM without serum and growth factors, and supplemented with lactate (10 mM) or not. Images of migrating cells were captured at the migration front around the coverslip margin. For quantification, BAECs were prestained with calcein and 50,000 cells were seeded in the upper chamber of the Matrigel-coated insert plate of a Biocoat Tumor Invasion System (BD). Bottom wells contained EGM-MV without serum and growth factors, supplemented with lactate (10 mM), VEGF (10 ng/ml, Sanvertech), CHC (5 mM) and/or saline. Twenty-four hours later, cells on the underside of the Fluoroblock membranes were quantified by measuring fluorescence at 535 nm after an excitation at 485 nm. Detached membranes were also fixed in 4% paraformaldehyde for 10 minutes and processed for immunohistochemistry using anti-caveolin-1 antibodies.

### Endothelial tube formation and aortic ring angiogenesis assays

Capillary-like tube formation [63] and mouse aorta ring assays [35] were performed as previously described. Where indicated, inhibitors were present both in the gels and in the supernatants.

### *In vivo* experiments

All mice were from Elevage Janvier. Where indicated, vehicle or CHC (25 µmol in 200 µl, [64]) was daily injected i.p. We used RJ:NMRI mice and an existing protocol for Matrigel plug implantation [58]. The protocols used for intramuscular tumor growth and intravital microscopy have also been reported previously [65], [66]. LLc cells were injected into syngeneic male 6–8 week-old C57/Bl6 J mice and TLT cells into syngeneic male RJ:NMRI mice. Surgery and imaging were performed on anesthetized (ketamine/xylazine) animals. All *in vivo* experiments were performed with approval of Duke University Institutional Animal Care and Use Committee (specific approval ID for this study was A069-03-02) and by *Université catholique de Louvain* (UCL) authorities (*Comité d'Ethique Facultaire pour l'Experimentation Animale*, specific approval ID for this study was TUMETABO) according to national animal care regulations.

### Statistical analyses

Results are expressed as mean ± SEM. Student's *t* test and 1-way ANOVA (Tukey's post-hoc test) were used where convenient. *p*<0.05 was considered statistically significant.

## Supporting Information

Figure S1 **Lactate does not support EC survival through increased oxygen consumption and CHC is devoid of intrinsic toxicity.** (A) HUVEC death was determined with a NucleoCounter. Confluent cells were assayed after 4-h of culture in serum-free DMEM containing the indicated substrates. *p<0.05 compared to glucose; *n* = 3. (B) EPR oximetry was used to determine the rate of oxygen consumption by HUVECs in DMEM containing 4500 mg/L glucose ±10 mM lactate. Slopes calculated from linear regressions are not statistically different (−1.973±0.174 for glucose, −1.902±0.275 for glucose+lactate, *p* = 0.84, *n* = 3). (A and B) Error bars reflect mean ± SEM.
(PDF)

Figure S2 **Lactate induces the nuclear translocation of HIF-1α in HUVECs.** HIF-1α protein expression was detected by Western blot in the cytosol (laminin A/C-negative fraction, 8 µg of total protein per well) and nucleus (laminin A/C-positive fraction, 10 µg of total protein per well) of HUVECs after a 24-h treatment with hypoxia or lactate as indicated. Densitometric analyses of HIF-1α protein expression (% of control) are provided below the corresponding bands; the experiment was repeated twice with similar results.
(PDF)

Figure S3 **Membrane expression of VEGFR2 and MCT1 in ECs.** (A) HUVECs were treated during 24 h with 10 mM lactate or not, after which the cells were immunostained with an antibody raised against an epitope comprising an extracellular domain of VEGFR2. Nuclei were stained with DAPI. Shown are representative pictures indicating that lactate stimulates the membrane expression of VEGFR2 in normoxic ECs. Omission of primary antibodies was used for control. (bar = 20 µm). (B) HUVECs were immunostained using antibodies against MCT1, its chaperon protein CD147, and CD31, and nuclei were stained with DAPI. Shown are representative pictures, including a merged picture, indicating that all 3 proteins colocalize at the cell membrane. Omission of primary antibodies was used for control. (bar = 20 µm).
(PDF)

Figure S4 **Inhibition of lactate-induced HIF-1α expression by 2-oxoglutarate, oxamate and LDH-B silencing.** Representative western blots are shown. (A and B) HIF-1α and β-actin protein expressions were detected in BAECs treated during 24-h as indicated with (A) 10 mM lactate and increasing concentrations of the PDH substrate 2-oxoglutarate (2-OG), (B) 10 mM lactate and/or 10 mM oxamate. (C) HUVECs were transfected with a specific siRNA against *LDH-B* or with a scrambled siRNA, and treated during 24-h with 20 mM lactate where indicated. The representative western blot shows HIF-1α, LDH-B and β-actin protein expressions.
(PDF)

Figure S5 **Protein extinctions in HUVECs after siRNA/shRNA delivery.** Representative western blots and quantification graphs are shown for HUVECs transfected with (A) a specific siRNA against *LDH-B* or a control siRNA (*n* = 2), or (B) a specific shRNA against *MCT1* or a control shRNA. *n* = 3. (A and B) Error bars reflect mean ± SEM.
(PDF)

Figure S6 **CHC is devoid of intrinsic toxicity for ECs.** HUVEC death was determined with a NucleoCounter. Confluent cells were assayed after 24-h culture in EBM2-MV containing glucose and serum and supplemented with lactate or CHC as indicated. *ns*, non-significant; *n* = 4. Error bars reflect mean ± SEM.
(PDF)

Figure S7 **Various MCT1 inhibitors block lactate-induced HIF-1α protein expression in normoxic ECs.** BAECs were treated during 24-h with 10 mM lactate and the MCT1 inhibitors indicated. *p<0.05, **p<0.01 compared to control; ^##^ *p*<0.01, ^###^ *p*<0.005 compared to lactate alone; *n* = 4. Error bars reflect mean ± SEM. NPPB, 5-nitro-2-[3-phenylpropylamino]-benzoate.
(PDF)

Figure S8 **MCT1 modulates basal and lactate-induced angiogenesis.** HUVECs transfected with a shRNA against MCT1 (target extinction = 86.7±3.3%) or with a scrambled (Scr) shRNA were seeded on undiluted growth factor-reduced Matrigel containing 10 mM lactate or not and monitored for their ability to generate vascular tubes. Shown are representative pictures (*n* = 3) captured after 12-h of culture. Bar = 1 mm.
(PDF)

Table S1 **Genes activated by lactate in normoxic ECs (RT2 Profiler PCR Array).**
(PDF)
