# Supplementary figures and images for: Targeting the Lactate Transporter MCT1 in Endothelial Cells Inhibits Lactate-Induced HIF-1 Activation and Tumor Angiogenesis
Source: PLoS One. 2012 Mar 13;7(3):e33418. doi: 10.1371/journal.pone.0033418 (PMC3302812; doi:10.1371/journal.pone.0033418)

Figure S1

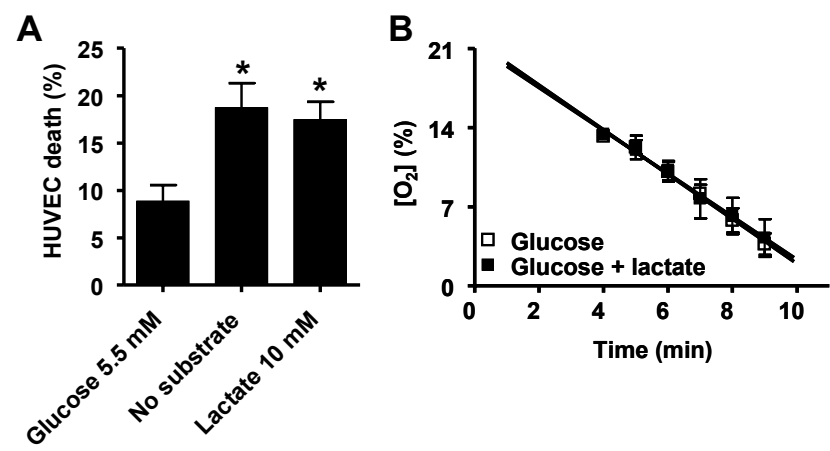

Supplement: Figure S1 — Lactate does not support EC survival through increased oxygen consumption and CHC is devoid of intrinsic toxicity. (A) HUVEC death was determined with a NucleoCounter. Confluent cells were assayed after 4-h of culture in serum-free DMEM containing the indicated substrates. *p<0.05 compared to glucose; n = 3. (B) EPR oximetry was used to determine the rate of oxygen consumption by HUVECs in DMEM containing 4500 mg/L glucose ±10 mM lactate. Slopes calculated from linear regressions are not statistically different (−1.973±0.174 for glucose, −1.902±0.275 for glucose+lactate, p = 0.84, n = 3). (A and B) Error bars reflect mean ± SEM. (PDF) [file pone.0033418.s001.pdf]

Figure S2

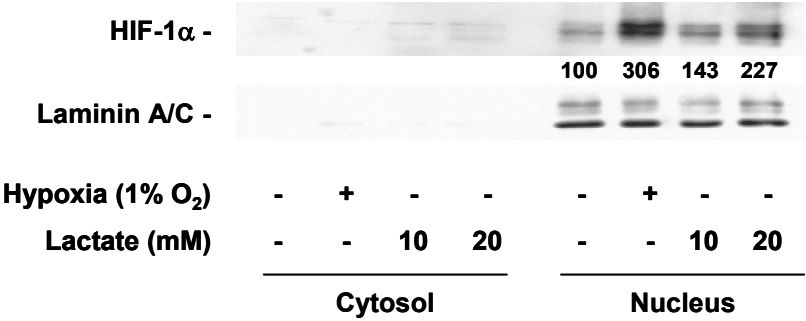

Supplement: Figure S2 — Lactate induces the nuclear translocation of HIF-1α in HUVECs. HIF-1α protein expression was detected by Western blot in the cytosol (laminin A/C-negative fraction, 8 µg of total protein per well) and nucleus (laminin A/C-positive fraction, 10 µg of total protein per well) of HUVECs after a 24-h treatment with hypoxia or lactate as indicated. Densitometric analyses of HIF-1α protein expression (% of control) are provided below the corresponding bands; the experiment was repeated twice with similar results. (PDF) [file pone.0033418.s002.pdf]

Figure S3

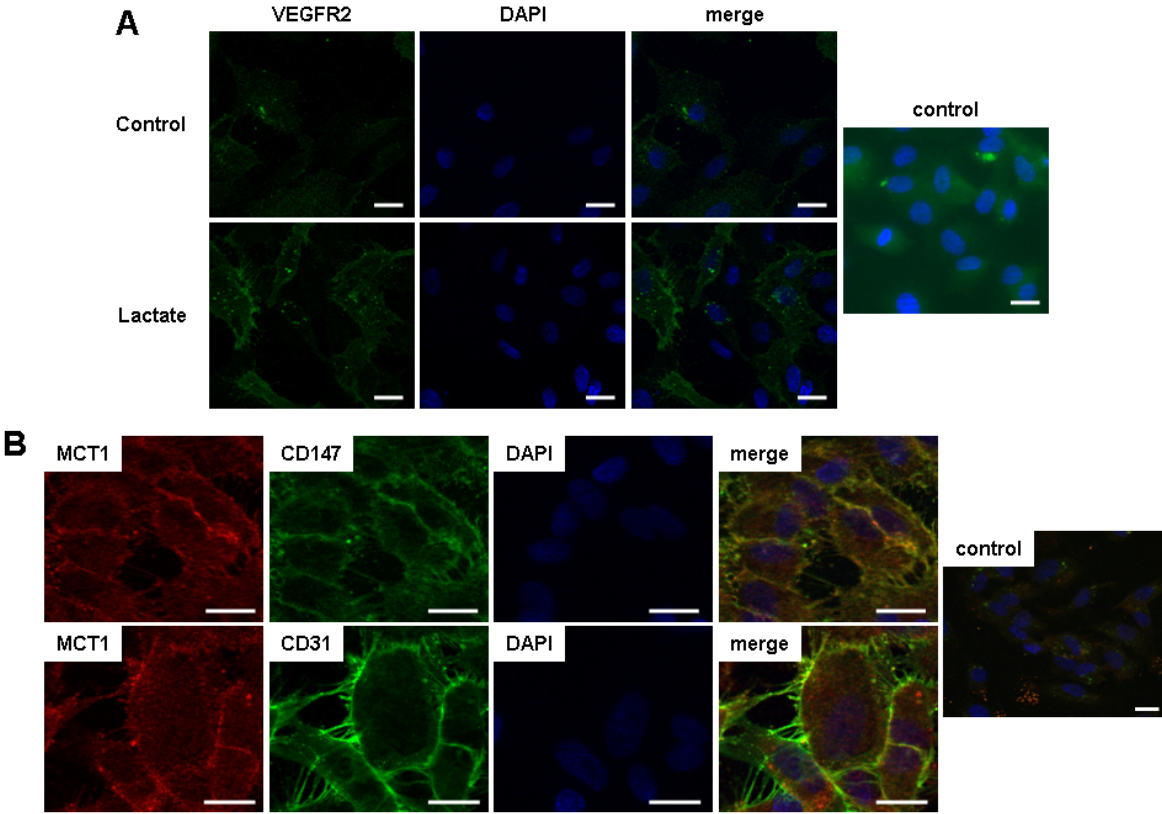

Supplement: Figure S3 — Membrane expression of VEGFR2 and MCT1 in ECs. (A) HUVECs were treated during 24 h with 10 mM lactate or not, after which the cells were immunostained with an antibody raised against an epitope comprising an extracellular domain of VEGFR2. Nuclei were stained with DAPI. Shown are representative pictures indicating that lactate stimulates the membrane expression of VEGFR2 in normoxic ECs. Omission of primary antibodies was used for control. (bar = 20 µm). (B) HUVECs were immunostained using antibodies against MCT1, its chaperon protein CD147, and CD31, and nuclei were stained with DAPI. Shown are representative pictures, including a merged picture, indicating that all 3 proteins colocalize at the cell membrane. Omission of primary antibodies was used for control. (bar = 20 µm). (PDF) [file pone.0033418.s003.pdf]

Figure S4

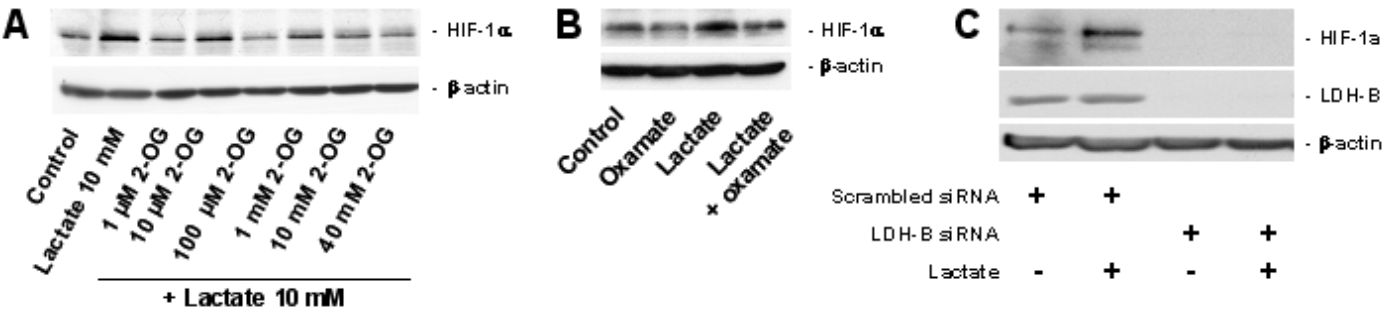

Supplement: Figure S4 — Inhibition of lactate-induced HIF-1α expression by 2-oxoglutarate, oxamate and LDH-B silencing. Representative western blots are shown. (A and B) HIF-1α and β-actin protein expressions were detected in BAECs treated during 24-h as indicated with (A) 10 mM lactate and increasing concentrations of the PDH substrate 2-oxoglutarate (2-OG), (B) 10 mM lactate and/or 10 mM oxamate. (C) HUVECs were transfected with a specific siRNA against LDH-B or with a scrambled siRNA, and treated during 24-h with 20 mM lactate where indicated. The representative western blot shows HIF-1α, LDH-B and β-actin protein expressions. (PDF) [file pone.0033418.s004.pdf]

Figure S5

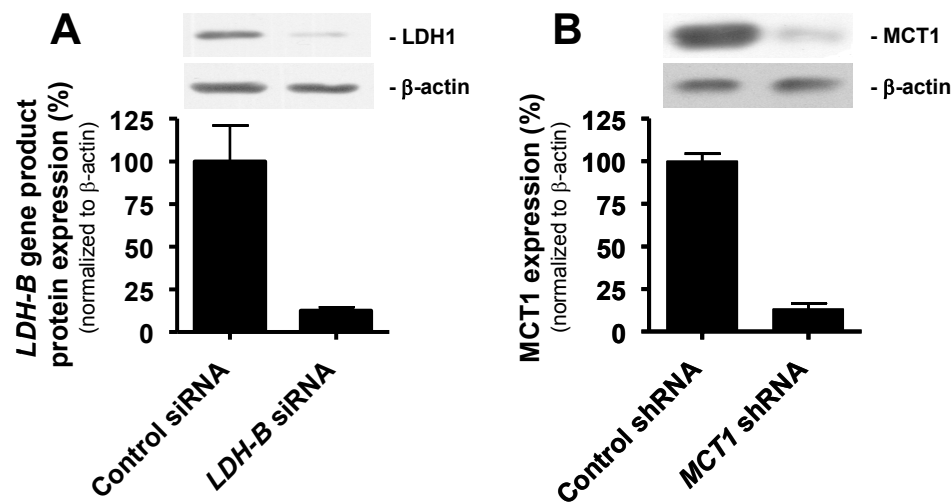

Supplement: Figure S5 — Protein extinctions in HUVECs after siRNA/shRNA delivery. Representative western blots and quantification graphs are shown for HUVECs transfected with (A) a specific siRNA against LDH-B or a control siRNA (n = 2), or (B) a specific shRNA against MCT1 or a control shRNA. n = 3. (A and B) Error bars reflect mean ± SEM. (PDF) [file pone.0033418.s005.pdf]

**Figure S6**

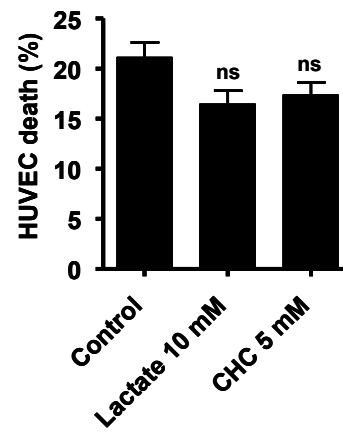

Supplement: Figure S6 — CHC is devoid of intrinsic toxicity for ECs. HUVEC death was determined with a NucleoCounter. Confluent cells were assayed after 24-h culture in EBM2-MV containing glucose and serum and supplemented with lactate or CHC as indicated. ns, non-significant; n = 4. Error bars reflect mean ± SEM. (PDF) [file pone.0033418.s006.pdf]

Figure S7

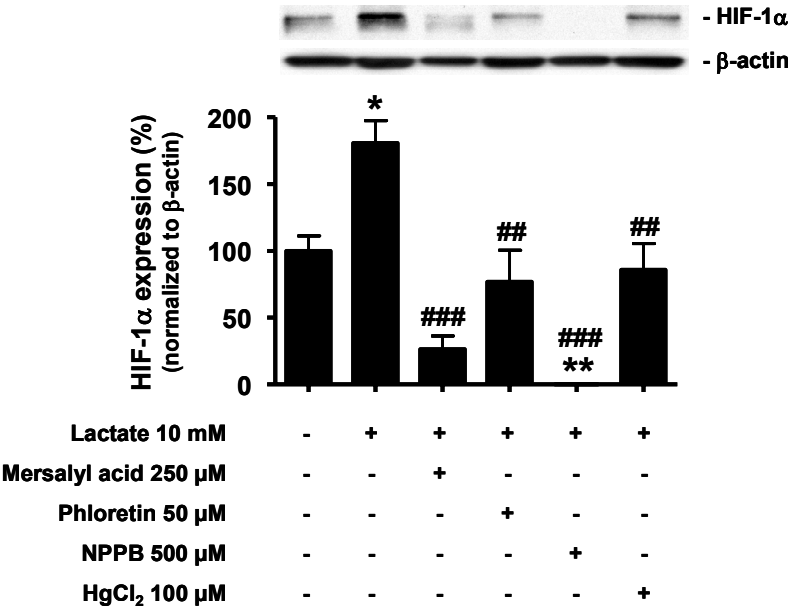

Supplement: Figure S7 — Various MCT1 inhibitors block lactate-induced HIF-1α protein expression in normoxic ECs. BAECs were treated during 24-h with 10 mM lactate and the MCT1 inhibitors indicated. *p<0.05, **p<0.01 compared to control; ## p<0.01, ### p<0.005 compared to lactate alone; n = 4. Error bars reflect mean ± SEM. NPPB, 5-nitro-2-[3-phenylpropylamino]-benzoate. (PDF) [file pone.0033418.s007.pdf]

Figure S8

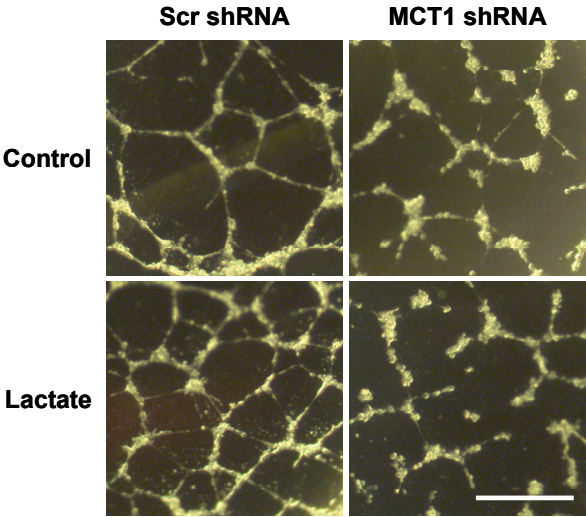

Supplement: Figure S8 — MCT1 modulates basal and lactate-induced angiogenesis. HUVECs transfected with a shRNA against MCT1 (target extinction = 86.7±3.3%) or with a scrambled (Scr) shRNA were seeded on undiluted growth factor-reduced Matrigel containing 10 mM lactate or not and monitored for their ability to generate vascular tubes. Shown are representative pictures (n = 3) captured after 12-h of culture. Bar = 1 mm. (PDF) [file pone.0033418.s008.pdf]
