# Supplementary material for: Targeting the Lactate Transporter MCT1 in Endothelial Cells Inhibits Lactate-Induced HIF-1 Activation and Tumor Angiogenesis
Source: PLoS One. 2012 Mar 13;7(3):e33418. doi: 10.1371/journal.pone.0033418 (PMC3302812; doi:10.1371/journal.pone.0033418)
Supplement: Table S1 — Genes activated by lactate in normoxic ECs (RT2 Profiler PCR Array). (PDF) [file pone.0033418.s009.pdf]

Table S1. Genes activated by lactate in normoxic ECs (RT2 Profiler PCR Array)[a]

Gene symbol	NCBI GeneID	Gene name	Fold induction	Relation to HIF-1	References
IPCEF1	26034	phosphoinositide-binding protein 3E	19.4	Unknown	-
MOCS3	27304	molybdenium cofactor synthesis 3	9.9	Unknown	-
RARA	5914	retinoic acid receptor α	7.6	Hypoxia-activated	1
SPTBN1	6711	spectrin β	6.6	Unknown	-
TH	7054	tyrosine hydroxylase	5.7	Target gene	2
CREBBP	1387	CREB binding protein	5.2	Transactivator	3
PDIA2	64714	protein disulfide isomerase	4.4	Hypoxia-activated	4
GNA11	2767	G protein α11	4.0	Unknown	-
EP300	2033	p300	3.5	Transactivator	3
DAPK3	1613	death-associated protein kinase 3	3.4	Unknown	-
CHGA	1113	chromogranin A	3.3	Unknown	-
NOS2A	4843	inducible NO synthase	3.0	Target gene	5
HIF1AN	55662	factor inhibiting HIF-1	2.9	Regulator	6
ANGPTL4	51129	angiopoietin-like 4	2.9	Hypoxia-induced	7
MT3	4504	metallothionein 3	2.9	Potential target gene<br>Regulator	8<br>9
NOTCH1	4851	NOTCH1	2.7	Target gene	10
HMOX1	3162	heme oxygenase 1	2.6	Target gene	5
HK2	3099	hexokinase 2	2.6	Target gene	5
VEGFA	7422	VEGF-A	2.5	Target gene	5
LEP	3952	leptin	2.5	Target gene	5
IL6ST	3572	IL6 signal transducer	2.3	Hypoxia-inducible	11
COL1A1	1277	Collagen type 1α1	2.2	Hypoxia-inducible	12
PPARA	5465	PPARα	2.1	Target gene	13
SSSCA1	10534	scleroderma autoantigen 1	2.1	Unknown	-
PRPF40A	55660	Fas ligand-associated factor 1	2.1	Unknown	-
SAE1	10055	SUMO1-activating enzyme 1	2.0	Unknown	-
EPAS1	2034	HIF-2α	2.0	Hypoxia-inducible	14
SLC2A1	6513	glucose transporter 1	2.0	Target gene	5
PRKAA1	5562	AMPK α1 catalytic subunit	1.9	Hypoxia-activated	15
IGF2	3481	insulin-like growth factor 2	1.9	Target gene	5
KAT5	10524	lysine acetyltransferase 5	1.9	Unknown	-

[a]Normoxic HUVECs were cultured during 24-h in the presence of 10 mM lactate or not. In both conditions, total RNA extracted from 3 independent dishes was pooled.

References:

1. Demary K, Wong L, Liou JS, Faller DV, Spanjaard RA (2001) Redox control of retinoic acid receptor activity: a novel mechanism for retinoic acid resistance in melanoma cells. *Endocrinology* 142:2600-2605.
2. Schnell PO, et al. (2003) Regulation of tyrosine hydroxylase promoter activity by the von Hippel-Lindau tumor suppressor protein and hypoxia-inducible transcription factors. *J Neurochem.* 85:483-491.
3. Ebert BL, Bunn HF (1998) Regulation of transcription by hypoxia requires a multiprotein complex that includes hypoxia-inducible factor 1, an adjacent transcription factor, and p300/CREB binding protein. *Mol Cell Biol* 18:4089-4096.
4. Chen SC et al. (2008) Acute hypoxia enhances proteins' S-nitrosylation in endothelial cells. *Biochem Biophys Res Commun* 377:1274-1278.
5. Semenza GL. Targeting HIF-1 for cancer therapy. *Nat Rev Cancer* 3:721-732.
6. Lando D, et al. (2002) FIH-1 is an asparaginyl hydroxylase enzyme that regulates the transcriptional activity of hypoxia-inducible factor. *Genes Dev*16:1466-1471.
7. Le Jan S, et al. (2003) Angiopoietin-like 4 is a proangiogenic factor produced during ischemia and in conventional renal cell carcinoma. *Am J Pathol* 162:1521-1528.
8. Wang B, Wood IS, Trayhurn P (2008) PCR arrays identify metallothionein-3 as a highly hypoxia-inducible gene in human adipocytes. *Biochem Biophys Res Commun* 368:88-93.
9. Kim HG, Hwang YP, Jeong HG (2008) Metallothionein-III induces HIF-1alpha-mediated VEGF expression in brain endothelial cells. *Biochem Biophys Res Commun* 369:666-671.
10. Bedogni B, Warneke JA, Nickoloff BJ, Giaccia AJ, Powell MB (2008) Notch1 is an effector of Akt and hypoxia in melanoma development. *J Clin Invest* 118:3660-3670.
11. Lam SY, Tipoe GL, Liong EC, Fung ML (2008) Chronic hypoxia upregulates the expression and function of proinflammatory cytokines in the rat carotid body. *Histochem Cell Biol* 130:549-559.
12. Falanga V, et al. (1993) Low oxygen tension increases mRNA levels of alpha 1 (I) procollagen in human dermal fibroblasts. *J Cell Physiol* 157:408-412.
13. Narravula S, Colgan SP (2001) Hypoxia-inducible factor 1-mediated inhibition of peroxisome proliferator-activated receptor alpha expression during hypoxia. *J Immunol* 166:7543-7548.
14. O'Rourke JF, Tian YM, Ratcliffe PJ, Pugh CW (1999) Oxygen-regulated and transactivating domains in endothelial PAS protein 1: comparison with hypoxia-inducible factor-1alpha. *J Biol Chem* 274:2060-2071.
15. Long YC, Zierath JR (2006) AMP-activated protein kinase signaling in metabolic regulation. *J Clin Invest* 116:1776-1783.
